# The Effect of Task Duration on Event-Based Prospective Memory: A Multinomial Modeling Approach

**DOI:** 10.3389/fpsyg.2017.01895

**Published:** 2017-11-01

**Authors:** Hongxia Zhang, Weihai Tang, Xiping Liu

**Affiliations:** ^1^School of Psychology, Beijing Normal University, Beijing, China; ^2^School of Education Science, Academy of Psychology and Behavior, Tianjin Normal University, Tianjin, China

**Keywords:** event-based prospective memory, prospective component, retrospective component, multinomial modeling, task duration

## Abstract

Remembering to perform an action when a specific event occurs is referred to as Event-Based Prospective Memory (EBPM). This study investigated how EBPM performance is affected by task duration by having university students (*n* = 223) perform an EBPM task that was embedded within an ongoing computer-based color-matching task. For this experiment, we separated the overall task’s duration into the filler task duration and the ongoing task duration. The filler task duration is the length of time between the intention and the beginning of the ongoing task, and the ongoing task duration is the length of time between the beginning of the ongoing task and the appearance of the first Prospective Memory (PM) cue. The filler task duration and ongoing task duration were further divided into three levels: 3, 6, and 9 min. Two factors were then orthogonally manipulated between-subjects using a multinomial processing tree model to separate the effects of different task durations on the two EBPM components. A mediation model was then created to verify whether task duration influences EBPM via self-reminding or discrimination. The results reveal three points. (1) Lengthening the duration of ongoing tasks had a negative effect on EBPM performance while lengthening the duration of the filler task had no significant effect on it. (2) As the filler task was lengthened, both the prospective and retrospective components show a decreasing and then increasing trend. Also, when the ongoing task duration was lengthened, the prospective component decreased while the retrospective component significantly increased. (3) The mediating effect of discrimination between the task duration and EBPM performance was significant. We concluded that different task durations influence EBPM performance through different components with discrimination being the mediator between task duration and EBPM performance.

## Introduction

Prospective memory (PM) is defined as remembering to perform an action in the future. There are two main types of PM according to their different cues: event-based prospective memory (EBPM) and time-based prospective memory (TBPM) (Einstein and McDaniel, 1990). Both EBPM and TBPM are necessary for daily life. EBPM is the remembering what one does when a certain target event occurs. For example, one remembers to buy milk when one passes by the supermarket. TBPM is involved when one has to perform an action in relation to time, such as returning a library book before a due date (Smith and Bayen, 2004). The current study focuses on EBPM.

Through EBPM studies, it has been identified that increasing the time between the encoding of information and the retrieval of that information has a negative effect on retrospective memory (RM) (Wixted and Ebbeson, 1991). To clarify, the prospective component is when one remembers what they must do and the retrospective component is when one remembers what one must do at a certain time (Einstein and McDaniel, 1996). For example, if the PM task is to remember to relay a message to colleague A, then remembering that this task must be done is the prospective component, whereas remembering to give the message when meeting colleague A is the retrospective component. In this experiment both components were studied separately.

Prospective memory shares some similarities with RM, but the results of many studies examining the effects of increased duration, between intention formation and the opportunity for retrieval, on PM performance are varied. Most studies confirmed that the time duration between intention and execution would have a negative effect or no effect on EBPM performance (Loftus, 1971; Einstein et al., 1992; Meier et al., 2006; Scullin et al., 2010a,b). However, there is a small but growing number of studies showing that longer task durations could increase individuals’ EBPM performance compared with shorter task durations (Hicks et al., 2000; Marsh et al., 2003; Martin et al., 2011). The effect of duration on these aspects is our study’s focus.

There were two significant considerations made when designing this study. The first is which task manipulations were suitable. Various filler tasks can be used during the period between the formation of an intention and the ongoing task (Einstein and McDaniel, 1990, 1996; Einstein et al., 2000). However, because this study needed to disentangle the components of PM, since it inherently involves both prospective and retrospective components, two methods were considered: The multinomial processing tree (MPT) model (Smith and Bayen, 2004) and the traditional accuracy measure (Cohen et al., 2001, 2003). The current study adopts the MPT model due to the limitations of the latter method^1^.

Smith and Bayen (2004) was the first study using the MPT model to distinguish the two components of EBPM using the preparatory attentional and memory processes (PAM) theory (Smith, 2003). Their study proposed that successful EBPM requires capacity-consuming preparatory processes which maintain a state of readiness to perform a task, and, without preparatory attentional processes, EBPM tasks cannot be successful. These processes make up the prospective component of PM, while the retrospective component is characterized as the discrimination between the PM targets and the non-targets, and as the recollection of the intended action (Smith, 2003; Smith and Bayen, 2004; Smith et al., 2007).

Current theories of EBPM suggest that the prospective component is resource-demanding (Smith, 2003, 2008). The PAM view suggests that under some circumstances PM can rely on either spontaneous retrieval processes or strategic monitoring processes for cue detection, depending on different tasks (McDaniel and Einstein, 2000; Einstein and McDaniel, 2005; Guynn, 2008; Cona et al., 2012). In focal PM tasks, the ongoing task involves processing the defining features of the PM cue (Einstein and McDaniel, 1990) while non-focal PM tasks are those in which the PM cue is not part of the information being extracted to continue the ongoing task (Park et al., 1997). Because it is suggested that non-focal tasks rely on resource-demanding processes (Einstein and McDaniel, 2005), to apply the MPT model, this study used a color-matching task, which is non-focal.

The second significant consideration when designing this study was the influence of filler and ongoing task durations on EBPM performance. In terms of measurements, the filler task duration is defined as the delay between the beginning of the ongoing task and the first PM target appearance is the ongoing task duration (Marsh et al., 2003; Martin et al., 2011). Some research used the filler task duration (Kvavilashvili, 1998; Guynn et al., 1998; Meier et al., 2006) while others used the ongoing task duration (Loftus, 1971). To account for this, we considered both the filler and ongoing task durations.

In Martin et al. (2011) which separated the two task durations and then observed their influence on EBPM performance independently, they found that in filler tasks EBPM performance for tasks lasting 3 min was better than for tasks lasting 18 min, while the opposite was true for the ongoing task. These results were thought to be caused by the longer filler task duration offering more opportunities for participants to self-remind, but this study showed that longer ongoing task durations would have made them more tired and thus reduced their ability to discriminate. We were also unable to find similar tendencies in our experiment using the same two levels of independent variables they manipulated. Furthermore, this study found no support for longer duration improving performance and suggests that further research should be conducted to clarify this issue.

Lastly, our overall aim is to further explore how the duration of the filler and the ongoing task affect the two components of EBPM, and to further explain how the tendencies of EBPM performance act as an underlying condition of prolonged task duration. In addition, we specifically attempted to determine whether a longer filler task duration could increase self-reminding and whether longer ongoing task duration would impair target discriminability.

## Materials and Methods

### Participants

The samples included 223 university students (106 female, age *M* = 20.90, *SD* = 1.63, ranged 18–24 years) who were native speakers of Chinese. They were randomly recruited to participate in this study. Exclusion criteria included current mental and physical health problems, color blindness, and successful task performance of the ongoing task under 50%. Participants were compensated for their participation.

### Design

**FIGURE 1:**
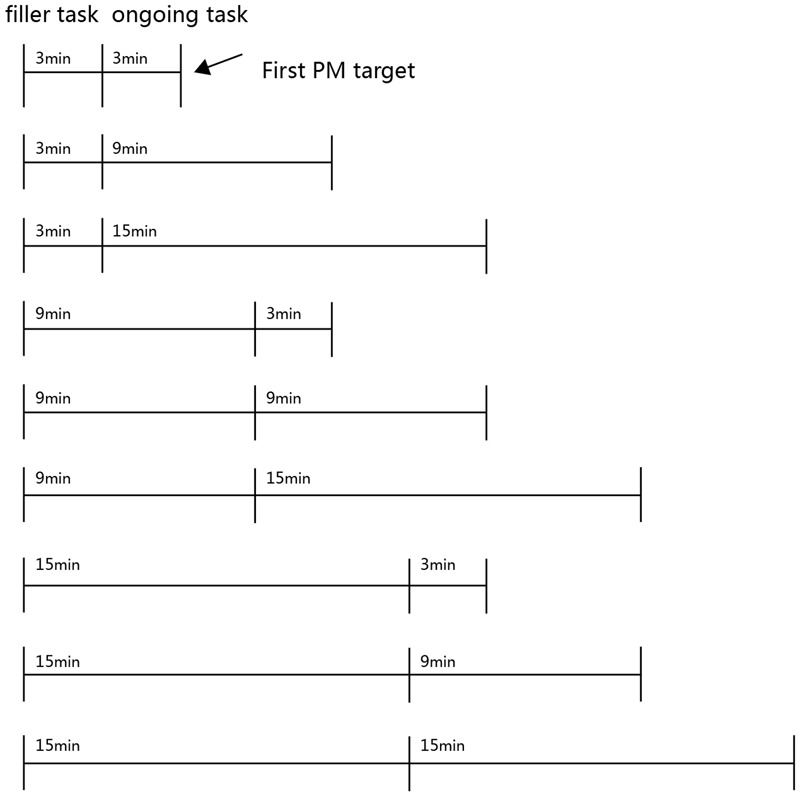
Schema of the design for the experiment.

The current experiment was a 3 (filler task duration: 3 min/9 min/15 min) × 3 (ongoing task: 3 min/9 min/15 min) between subjects-design. The number and age of participants in each condition are depicted in **Table 1**. The filler task duration was defined as the length of time from the intention to the beginning of the ongoing task, which was performed for 3, 9, or 15 min. The ongoing task duration was defined as the length of time from the beginning of the ongoing task to the appearance of the first PM cue. The dependent variables were PM performance, the prospective and retrospective components that were measured based on the multinomial modeling parameters. The schema of the experimental design is shown in **Figure 1**.

**Table 1:** The number and age of participants in each conditions.

Condition	Age		*N*
	*M*	*SD*	
Ft (3 min)/ot (3 min)	21.23	1.68	29
Ft (3 min)/ot (9 min)	20.36	1.65	33
Ft (3 min)/ot (15 min)	21.59	1.26	22
Ft (9 min)/ot (3 min)	20.56	1.55	29
Ft (9 min)/ot (9 min)	21.04	1.77	25
Ft (9 min)/ot (15min)	20.63	1.61	24
Ft (15 min)/ot (3 min)	21.14	1.65	21
Ft (15 min)/ot (9 min)	20.58	1.57	19
Ft (15 min)/ot (15 min)	21.29	1.68	21

*Ft, filler task; ot, ongoing task.*

### Materials

In the filler task, participants were required to complete a word frequency judgment activity. The materials for the filler task were composed of 90 low frequency words that were selected from *The China People’s Daily*. In the ongoing task, participants were required to complete a color-matching activity. The stimuli of this task were colored rectangles (red, blue, green, yellow, or white) and 300 medium frequency words selected from *The China People’s Daily*. The words *frog* and *tortoise* were used as EBPM targets.

### Multinomial Processing Tree (MPT) Model

The MPT model assumes that participants are undertaking discrete cognitive processes during task performance (Smith and Bayen, 2004). In the experiments presented here, we used a color-matching task to illustrate the MPT model of EBPM. For this task, there were four different trial types: (1) The PM target and the color matches, (2) the PM target but the color does not match, (3) there is no PM target but the color matches, and (4) there is no PM target and the color does not match. Each participant had three response options: “Match,” “Non-match,” and “PM” in each trial.

**FIGURE 2:**
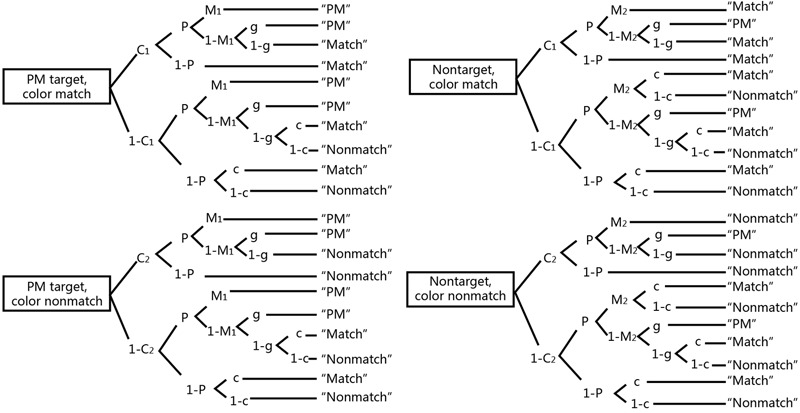
Multinomial model of EBPM (Smith and Bayen, 2004). *P* = probability of engaging in preparatory attentional processes; *M* = probability of discriminating between targets and non-targets; *C*_1_ = probability of detecting a color match; *C*_2_ = probability of detecting a color does not match; *g* = probability of guessing a PM target; *c* = probability of guessing a color matches.

As seen in **Figure 2**, the top portion of the first tree represents the target words on match trials to illustrate the cognitive process. This is a PM target presented in a match trial. *C*_1_ is the probability of detecting color matches and (1-*C*_1_) is the probability of failing to detect color matches. *P* is the probability that one will engage in preparatory attentional processes (i.e., the prospective component) and the probability of an incorrect response is (1-*P*).

If the participant can detect color matches (*C*_1_) and engage in preparatory processes (*P*), he or she may either recognize the word as the PM target (*M*_1_) resulting in a “PM” response or not (1-*M*_1_). If they do not recognize the word, they can either guess that it is a target (*g*), resulting in a “PM” response, or not (1-*g*), resulting in a “Match” response. If the participant can detect color matches (*C*_1_) but does not engage in preparatory processes (1-*P*), it will result in a “Match” response. If the participant does not detect color matches (1-*C*_1_) and engages in preparatory processes (*P*), he or she may either recognize the word as PM target (*M*_1_) resulting in a “PM” response or not (1-*M*_1_). If they do not recognize the word, they can either guess that it is a target (*g*), resulting in a “PM” response, or not (1-*g*), then they may guess that the color matches *c*, resulting in a “Match” response, or not (1-*c*), resulting in a “Non-match” response.

**FIGURE 3:**
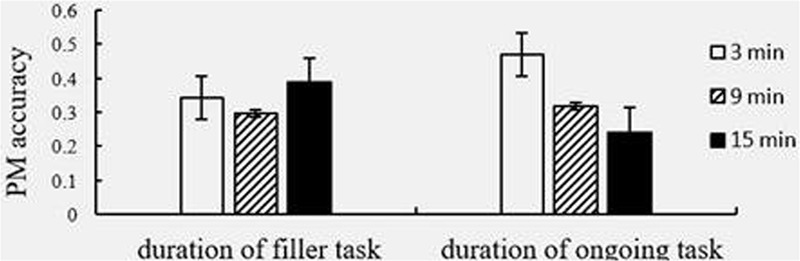
Proportion of correct event-based prospective memory (EBPM) performance by duration of filler task and ongoing task. Error bars represent 95% confidence intervals.

The model as illustrated in **Figure 2** has seven parameters (*C*_1_, *C*_2_, *P, M*_1_, *M*_2_, *g*, *c*), while there are only four equations created by summing branch probabilities for the tree in **Figure 3** for each response. A necessary condition for the global identifiability of a model is that the number of the parameters not be greater than the number of the equations of possible response-category probabilities. So we should set the constraints on model parameters. These constraints may be either constraints that set parameters to certain predetermined values (Erdfelder and Buchner, 1998) or equality constraints setting two (or more) parameters equal to each other (see, e.g., Batchelder and Riefer, 1999; Bayen et al., 1996). Constraints on the model parameter led to an identifiable and testable four- parameter submodel (*P*, *M*, *C_1_*, *C_2_*). We set *M*_1_ = *M*_2_, *c* = 0.5, *g* = 0.1 (see Smith and Bayen, 2004, 2006), resulting in a model with four free parameters: *P*, *M*, *C*_1_, and *C*_2_.

### Procedure

The experiment was divided into four stages: the practice stage, the filler task, the ongoing task with embedded EBPM targets, and the questionnaire stage. All participants were first given the instructions for the ongoing task. Participants then performed a practice phase of the ongoing task until the task was understood. Upon completion of the practice phase, participants in the EBPM condition were presented with the EBPM instruction to press the letter B when they encountered the word *frog* or *tortoise* during the next phase of the ongoing task. After the practice phase of the ongoing task and the instruction of the PM task, participants were presented with instructions for the filler task, and were instructed to perform this task before the ongoing task. Once the filler task ended, there was an 8 s break which participants could use to prepare for the next task.

In the filler task, participants were required to perform a continuous episodic frequency judgment task. Ninety low frequency words were presented for 1500 ms once per trial with random replacement. Participants were then instructed to record how many times they saw each word.

We used a color-matching task for the ongoing task (Smith and Bayen, 2004). In the color-matching task, participants saw four colored rectangles (red, blue, green, yellow, or white) followed by a word displayed in one of the five colors in each trial. The rectangles were displayed on the screen for 500 ms per trial with a 250 ms interstimulus interval. Participants were then asked to judge whether the color of the word matched one of the colors shown in the preceding set as quickly and accurately as possible. Matched responses were indicated with the F key and non-matched responses were indicated with the J key. Half of the trials were match trials and the other half were non-match.

In this study, when participants gave a PM task response, the PM response was given instead of the ongoing task response. The orders of match and non-match trials were random, and the colors were counterbalanced so an equal amount of each was presented within the trials. Also, the first EBPM cue would appear at 3, 9, or 15 min from the beginning of the ongoing task, and the second EBPM cue would appear 2 min after the presentation of the first EBPM cue. Lastly, the ongoing task finished 2 min after the appearance of the second EBPM cue.

After task completion, participants would be asked to complete an interview consisting of the three following questions. (1) Please recall your level of self-reminding for the EBPM cue on the record task (remembering to press the B key), and rate it on a 1–5 scale. (2) Please recall your level of self-reminding for the EBPM cue on the color-matching task (remembering to press B), and rate it on a 1–5 scale. (3) Please judge your level of discriminability before the EBPM cue appeared, and rate it on a 1–5 scale.

## Results

### Event-Based Prospective Memory (EBPM) Performance

Event-based prospective memory performance was measured by accuracy and reaction time (RT). Accuracy is the proportion of the EBPM target correctly selected by participants when the two EBPM cues were presented during the ongoing task. In dealing with the accuracy and RT, the trials which outliers outside *M* ± 3*SD* have been excluded.

As shown in **Figure 3**, we found a declining and then increasing trend of EBPM performance when the duration of the filler task increased. In addition, EBPM performance declined sharply when the duration of the ongoing task was lengthened. We used a 3 (duration of filler task) × 3 (duration of ongoing task) between-subjects ANOVA to analyze the accuracy and RT of EBPM. The results of analyzing accuracy showed that the effect of changing filler task duration was not statistically significant, *F*_(2,214)_ = 0.935, *p* = 0.394, η^2^ = 0.009. However, the effect of changing ongoing task duration was significant, *F*_(2,214)_ = 7.330, *p* = 0.001, η^2^ = 0.064. *Post hoc* comparisons showed that the EBPM performance at 3 min within the ongoing task was significantly higher than at 9 min (*p* = 0.007) and at 15 min (*p* < 0.0001), and there was no significant difference between 9 and 15 min (*p* = 0.342). The interaction of these two durations was also not significant, *F*_(2,214)_ = 1.842, *p* = 0.122, η^2^ = 0.033, and the RT results showed that the effect of filler task duration was not statistically significant either, *F*_(2,214)_ = 1.225, *p* = 0.296, η^2^ = 0.011. Lastly, the effect of ongoing task duration did not appear significant, *F*_(2,214)_ = 0.439, *p* = 0.645, η^2^ = 0.004; nor was the interaction of these two durations, *F*_(2,214)_ = 1.430, *p* = 0.225, η^2^ = 0.033.

### Multinomial Modeling Results

We used Multitree programs to analyze the MPT model data, using goodness-of-fit statistics, and parameter estimates for MPT models (Moshagen, 2010).

#### Model Fit

Goodness-of-fit tests with log-likelihood (*G*^2^) were used to predict the multinomial model fit, which was asymptotically χ^2^-distributed (Hu and Batchelder, 1994). Both the model fit and the parameter values were obtained with available software (Stahl and Klauer, 2007; Moshagen, 2010).

Goodness-of-fit of the models was evaluated for the complete data of each condition (i.e., 3 filler tasks × 3 ongoing tasks, resulting in 36 trees) using the likelihood-ratio statistic *G*^2^, which is asymptotically chi-square distributed (with *df* = 68). The model fit the data well, *G*^2^ (68) = 42.25, *p* = 0.76. The data of the model fit for both the filler task and the ongoing task was shown on **Table 2**.

**Table 2:** Values for tests of goodness-of-fit.

Type of delay	*G*^2^(4)	*p*
Ft (3 min)	5.69	0.22
Ft (9 min)	2.10	0.51
Ft (15 min)	2.32	0.68
Ot (3 min)	2.40	0.66
Ot (9 min)	4.48	0.35
Ot (15 min)	1.47	0.75

*Critical Δ*G*^*2*^ = 9.46.*

#### Parameter Estimates

**Table 3** shows the estimates of the four free model parameters across the three durations and the different task types. To examine potential differences in each parameter across durations in the filler tasks and ongoing tasks, we used significance tests for each parameter by setting the value of a given parameter as equal for the two conditions and evaluating the change in the fit of the model. If this constraint reduces the fit of the model significantly [*G*^2^(1) > 3.84], it means that the two conditions differ significantly in the estimates of the parameter.

**Table 3:** Parameter estimates across duration in different task types.

Condition	*P*	*M*	*C_1_*	*C_2_*
Ft (3 min)	0.36	0.97	0.52	0.78
	(0.29–0.43)	(0.96–0.99)	(0.50–0.54)	(0.77–0.79)
Ft (9 min)	0.29	0.96	0.57	0.79
	(0.21–0.36)	(0.94–0.98)	(0.55–0.59)	(0.78–0.80)
Ft (15 min)	0.47	0.99	0.56	0.80
	(0.38–0.55)	(0.99–1.00)	(0.54–0.58)	(0.79–0.81)
Ot (3 min)	0.46	0.96	0.56	0.73
	(0.38–0.54)	(0.95–0.97)	(0.53–0.58)	(0.71–0.75)
Ot (9 min)	0.30	0.97	0.46	0.78
	(0.23–0.38)	(0.96–0.99)	(0.44–0.48)	(0.76–0.79)
Ot (15 min)	0.43	0.99	0.61	0.83
	(0.26–0.42)	(0.98–1.00)	(0.59–0.62)	(0.81–0.84)

**FIGURE 4:**
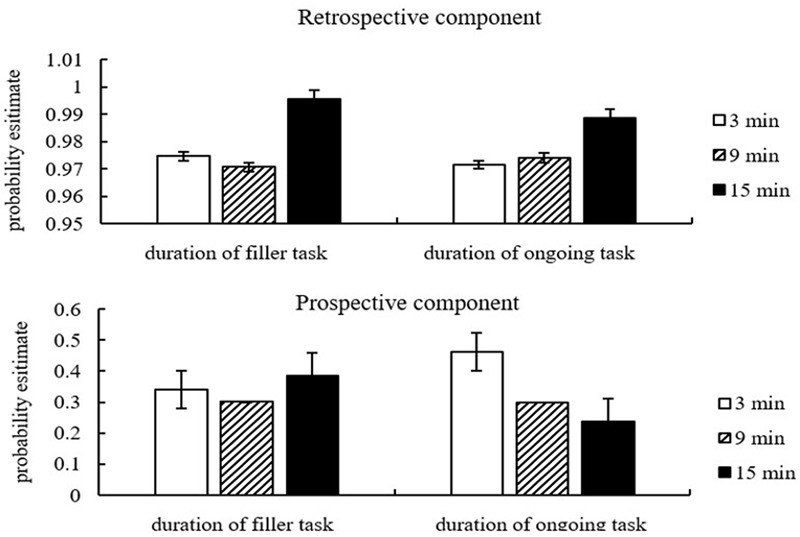
Parameter estimates for different levels of filler task and ongoing task. Error bars represent 95% confidence intervals.

**Figure 4** and **Tables 3, 4** show that parameters *P* and *M* did not change significantly as the duration of the filler task increased from 3 to 9 min, but they increased significantly as the duration of the filler task increased from 9 to 15 min. Parameter *P* decreased significantly as the duration of the ongoing task increased from 3 to 9 min, but there was no significant difference when the duration of ongoing task increased from 9 to 15 min. There was also no significant difference in Parameter M when the ongoing task duration increased from 3 to 9 min, but there was a significant increase as the ongoing task duration increased from 9 to 15 min.

**Table 4:** Δ*G*^2^ values testing task type and duration differences.

Comparison	*P*	*M*	*C_1_*	*C_2_*
Ft (3 min) vs. ft (9 min)	3.38	0.291	27.675^∗∗∗^	2.260
Ft (3 min) vs. ft (15 min)	7.36^∗∗^	24.89^∗∗∗^	20.36^∗∗^	9.06^∗^
Ft (9 min) vs. ft (15 min)	17.17^∗∗∗^	19.74^∗∗∗^	1.03	1.45
Ot (3 min) vs. ot (9 min)	16.16^∗∗∗^	0.19	107.31^∗∗∗^	47.50^∗∗∗^
Ot (3 min) vs. ot (15 min)	7.50^∗∗^	9.18^∗^	45.70^∗∗∗^	270.26^∗∗∗^
Ot (9 min) vs. ot (15 min)	1.23	4.77^∗^	337.54 ^∗∗∗^	51.28^∗∗∗^

*Δ*G*^*2*^ = increment of the chi-square distributed fit statistic (*df* = 1) compared with the unrestricted model; Critical *G*^*2*^(1) = 3.84. ^∗^*p* < 0.05; ^∗∗^*p* < 0.01; ^∗∗∗^*p* < 0.001.*

#### Ongoing Task Parameters

The ability to detect color matches *C*_1_ and detect color non-matches *C*_2_ are ongoing task parameters. As seen in **Tables 3, 4**, *C*_1_ increased significantly as the duration of the filler task increased from 3 to 9 min, but there was no significant change as the duration of filler task increased from 9 to 15 min. When the ongoing task duration increased from 3 to 15 min, there was a significant trend of increasing before decreasing. *C*_2_ did not change significantly as the duration of the filler task increased from 3 to 9 min and from 9 to 15 min, but it increased significantly as the duration of filler task increased from 3 to 15 min. Lastly, as the ongoing task duration increased from 3 to 15 min, *C*_2_ continued to increase significantly.

### Relevance between the Filler Task Duration and the EBPM Performance – The Mediator Effect of Self-reminding

**FIGURE 5:**
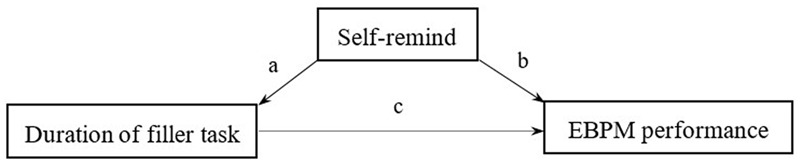
The mediating effect by self-reminding between duration of filler task and EBPM performance.

We built the model (**Figure 5**) under the assumption of self-reminding being the mediator between filler task duration and EBPM performance.

The results of analyzing the mediating effect show that the extent of self-reminding increased as the filler task duration increased, though it did not improve EBPM performance. Also, the Sobel test showed that self-reminding was not the mediator between the duration of the filler task and EBPM performance (see **Table 5**).

**Table 5:** Test of the mediating effect (self-reminding, *w*).

	Standardized regression equation	Regression coefficient test
First step	*Y* = 0.045X	*SE* = 0.04, *t* = 0.60
Second step	*W* = 0.019X	*SE* = 0.12, *t* = 0.26
Third step	*Y* = 0.35X	*SE* = 0.03, *t* = 0.54
	+0.54W	*SE* = 0.02, *t* = 8.36^∗∗^

*^∗∗^*p* < 0.01.*

### Relevance between Ongoing Task Duration and EBPM Performance – The Mediator Effect of Self-reminding and Discriminability

**FIGURE 6:**
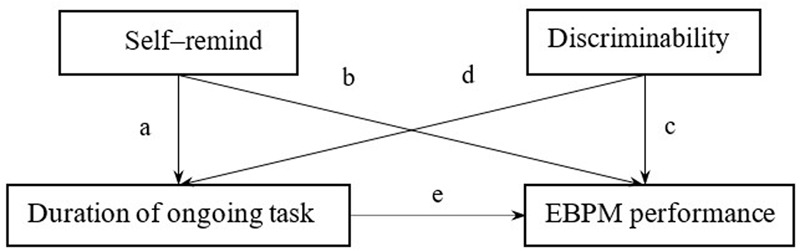
The mediating effect by self-reminding and discriminability between duration of filler task and EBPM performance.

We built the model (see **Figure 6**) under the assumption that self-reminding and discriminability were the mediators between the duration of the ongoing task and EBPM performance.

Results from the Sobel test showed that discriminability was the mediator between the duration of the ongoing task and EBPM performance, *z* = -1.65, *p* = 0.052 (marginally significant), while self-reminding was not, *z* = -1.14, *p* = 0.072 (**Table 6**).

**Table 6:** Test of the mediating effect (self-reminding, *w*_1_; discriminability, *w*_2_).

	Standardized regression equation	Regression coefficient test
First step	*Y* = -2.1X	*SE* = 0.04, *t* = -2.8^∗^
Second step	*W*_1_ = 0.134X	*SE* = 0.12, *t* = -1.79
	*W*_2_ = -0.18X	*SE* = 0.12, *t* = -2.41^∗^
Third step	*Y* = -0.107X	*SE* = 0.02, *t* = -1.75
	+0.46 W_1_	*SE* = 0.02, *t* = 6.90^∗∗^
	+0.22 W_2_	*SE* = 0.02, *t* = 8.36^∗∗^

*^∗^*p* < 0.05; ^∗∗^*p* < 0.01.*

## Discussion

The aim of the present study was to explore how the duration of different tasks affect EBPM performance, to disentangle the prospective and retrospective components of EBPM, to dissociate the durations of different task effects through multinomial modeling, and to determine whether task duration influences EBPM via self-reminding or discrimination.

As predicted, our results were similar to those observed by Martin et al. (2011), that was, the filler task duration and the ongoing task duration affected EBPM performance differently. We used three duration levels for each of the two separated tasks in order to determine their effects on EBPM performance. The behavioral results showed that filler task duration did not affect EBPM performance significantly, but there was U-type tendency when prolonging the filler task duration. This was consistent with the results of previous studies but with greater detail (Hicks et al., 2000; Meier et al., 2006; Martin et al., 2011).

In the current study, EBPM performance decreased rapidly when the ongoing task duration increased. Previous studies reported that both PM and RM were affected by increasing the duration of the task, but they did not analyze these two task durations separately (Wixted and Ebbeson, 1991; Brandimonte and Passolunghi, 1994; Meier et al., 2006; Scullin et al., 2010a,b). From the findings of the current study, the highest EBPM performance was found after 15 min of the filler task and after 3 min of the ongoing task.

The MPT model was used to explore the change of EBPM processes associated with both of these two task durations. The model results showed that both the prospective and retrospective component had an increasing trend as the duration of the filler task increased. Also, the prospective component decreased while the retrospective component increased with the increase of the ongoing task duration. Therefore, we could infer that the cause of poor EBPM performance in the study was the decrease of the prospective component.

Based on PAM theory, we used a color-matching task in our study to serve as a non-focal task. Unlike focal tasks, non-focal tasks require attention resources continuously for the entire task (Scullin et al., 2010a,b). In addition, attentional resources for each task were limited. Therefore, attentional resources were consumed when the duration of the ongoing task was prolonged, which reduced the prospective component, thus resulting in a decline in PM performance (Smith and Bayen, 2005, 2006; Smith et al., 2011; Pavawalla et al., 2012).

According to this study’s results, participants showed an increased ability to discriminate between target and non-target events, as measured by parameter *M* (the retrospective component) in the longer duration ongoing task. One possible explanation was that there were more opportunities to engage in self-reminding during the long ongoing task. This hypothesis was verified by the mediation test, which will be further discussed in the following section. The model results also showed that the ability to detect color matches increased at the early stages of the long duration filler task and tended to decrease before increase as the duration of ongoing task increased. The ability to detect color non-matches improved during both the filler and ongoing tasks as the duration was increased which suggests that the participants continued to consume attention resources as the filler or ongoing task duration increased. As the ongoing task constantly demanded more attention, participants had fewer attentional resources to perform the PM task.

For the purpose of investigating the mediator effect of self-reminding and discrimination processes on EBPM performance, we assumed that these two factors were the mediator between the two task durations and the EBPM performance. The results of mediation analysis showed that self-reminding had no significant mediation effect on the two task durations nor on EBPM performance. However, there was a marginally significant mediation effect of discrimination on the ongoing task and on EBPM performance. These results were consistent with Martin et al. (2011) and supplemented them to some extent. Participants would remind themselves more frequently as the duration filler task increased, and self- reminding improved their performance.

The current study used elements from a study by Hicks et al. (2000), which manipulated three kinds of conditions to encourage people to self-remind: (1) A single 15 min filler task without a break, (2) five separate tasks lasting a total of 15 min with each task lasting for 3 min, and (3) a 15 s break in the single 15 min filler task every 3 min. The results suggested that the last method results in the best EBPM performance, and that the last condition was the best method for encouraging self-reminding. However, there was an issue that was overlooked by previous research: That self-reminding also occurred during an ongoing task.

In line with filler task duration, self-reminding increased quickly as ongoing task duration was prolonged. The results from further analysis indicated that it was not the decline of self-reminding that impaired EBPM performance. The current study also took discriminability into account as the results indicated that long ongoing task duration reduces EBPM performance by descending discriminability. This was in line with the results of MPT, which stated that both the discriminability and the prospective components consume attention resources.

In daily life, the EBPM task would not appear immediately after we recalled it, so this study went against participants’ natural EBPM performance. According to the results of the present study, EBPM performance declined quickly when the ongoing task duration increased, and it increased when the filler task duration increased. Based on these findings, we aimed to prevent the decline associated with the long duration of an ongoing task and maintain EBPM performance as good as possible by lengthening the filler task duration and shortening the ongoing task duration. Then, researchers were able to adjust participants’ distribution of attentional resources or expand the amount of attentional resources needed to maintain the prospective component during long durations of an ongoing task, especially in particular groups that might have poorer EBPM (Smith and Bayen, 2005, 2006; Smith et al., 2011; Pavawalla et al., 2012) performance than others (e.g., older people, brain-damaged patients, low working memory span people, etc.).

Some possible limitations of the present work were related to the tasks we used as the filler and the ongoing task (word frequency judgment vs. color) which could interfere with the result, even if the filler task and the ongoing task were different in many previous articles (Hicks et al., 2000; McDaniel and Einstein, 2000; Marsh et al., 2003; Einstein and McDaniel, 2005; Martin et al., 2011; Pavawalla et al., 2012). In future studies, we will take this interference into consideration.

## Ethics Statement

We submitted our research to Beijing Normal University and Tianjin Normal University ethics committee for review and both committees approved this study. All participants consented to involve this study.

## Author Contributions

HZ and WT were in charge of putting the problem, data collection and statistics and paper writing. XL was in charge of paper modification.

## Conflict of Interest Statement

The authors declare that the research was conducted in the absence of any commercial or financial relationships that could be construed as a potential conflict of interest. The reviewer GC and handling Editor declared their shared affiliation.

## Footnotes

1 Cohen et al. (2001) gave similar definitions to the above, but their study relies on cues: the prospective component relies upon being able to successfully detect the cue, and the retrospective component is recalling the correct response when the cue is detected. Based on these definitions, there is no PM process once a participant fails to detect the cue according to this definition. However, Smith (2003) showed that participants who had to perform an embedded EBPM task while also performing an ongoing task took between 200 and 300 ms longer than participants who performed the ongoing task alone. Other studies reached similar conclusions (Burgess et al., 2001; Marsh et al., 2003; Smith and Bayen, 2004; Smith et al., 2007; Smith, 2008). These show that the process of PM also exists even when participants execute ongoing tasks without recognizing the cues. Therefore, this study focuses solely on whether or not PM responses were made.

## References

[B1] Batchelder W. H. Riefer D. M. (1999). Theoretical and empirical review of multinomial process tree modeling. *Psychon. Bull. Rev.* 6 57–86. 10.3758/bf03210812 12199315

[B2] Bayen U. J. Murnane K. Erdfelder E. (1996). Source discrimination, item detection, and multinomial models of source monitoring. *J. Exp. Psychol. Learn. Mem. Cogn.* 22 197–215. 10.1037/0278-7393.22.1.197 20702864

[B3] Brandimonte M. A. Passolunghi M. C. (1994). The effect of cue familiarity, cue distinctiveness, and retention interval on prospective remembering. *Q. J. Exp. Psychol.* 47 565–587. 10.1080/14640749408401128 7938668

[B4] Burgess P. W. Quayle A. Frith C. D. (2001). Brain regions involved in prospective memory as determined by positron emission tomography. *Neuropsychologia* 29 545–555. 10.1016/S0028-3932(00)00149-4 11257280

[B5] Cohen A.-L. Dixon R. A. Lindsay D. S. Masson M. E. J. (2003). The effect of perceptual distinctiveness on the prospective and retrospective components of prospective memory for young and older adults. *Can. J. Exp. Psychol.* 57 274–289. 10.1037/h0087431 14710865

[B6] Cohen A.-L. West R. Craik F. I. M. (2001). Modulation of the prospective and retrospective components of memory for intentions in younger and older adults. *Aging Neuropsychol. Cogn.* 8 1–13. 10.1076/anec.8.1.1.845

[B7] Cona G. Arcara G. Tarantino V. Bisiacchi P. S. (2012). Electrophysiological correlates of strategic monitoring in event-based and time-based prospective memory. *PLOS ONE* 7:e31659. 10.1371/journal.pone.0031659 22363699 PMC3283681

[B8] Einstein G. O. Holland L. J. McDaniel M. A. Guynn M. J. (1992). Age-related deficits in prospective memory: the influence of task complexity. *Psychol. Aging* 7 471–478. 10.1037/0882-7974.7.3.471 1388869

[B9] Einstein G. O. McDaniel M. A. (1990). Normal aging and prospective memory. *J. Exp. Psychol. Learn. Mem. Cogn.* 16 717–726. 10.1037/0278-7393.16.4.717 2142956

[B10] Einstein G. O. McDaniel M. A. (1996). “Retrieval processes in prospective memory: theoretical approaches and some new empirical findings,” in *Prospective memory: Theory and applications* eds Brandimonte M. Einstein G. O. McDaniel M. A. (Mahwah, NJ: Erlbaum) 115–141.

[B11] Einstein G. O. McDaniel M. A. (2005). Prospective memory: multiple retrieval processes. *Curr. Dir. Psychol. Sci.* 14 286–290. 10.1111/j.0963-7214.2005.00382.x

[B12] Einstein G. O. McDaniel M. A. Manzi M. Cochran B. Baker M. (2000). Prospective memory and aging: forgetting intentions over short delays. *Psychol. Aging* 15 671–683. 10.1037/0882-7974.15.4.671 11144326

[B13] Erdfelder E. Buchner A. (1998). Decomposing the hindsight bias: a multinomial processing tree model for separating recollection and reconstruction in hindsight. *J. Exp. Psychol. Learn. Mem. Cogn.* 24 387–414. 10.1037/0278-7393.24.2.387

[B14] Guynn M. J. (2008). “Theory of monitoring in prospective memory: instantiating a retrieval mode and periodic target checking,” in *Prospective Memory: Cognitive, Neuroscience, Developmental, and Applied Perspectives* eds Kliegel M. McDaniel M. A. Einstein G. O. (New York: Lawrence Erlbaum Associates) 53–76.

[B15] Guynn M. J. McDaniel M. A. Einstein G. O. (1998). Prospective memory: when reminders fail. *Mem. Cognit.* 26 287–298. 10.3758/BF03201140 9584436

[B16] Hicks J. L. Marsh R. L. Russell E. J. (2000). The properties of retention intervals and their effect on retaining prospective memories. *J. Exp. Psychol. Learn. Mem. Cogn.* 26 1160–1169. 10.1037/0278-7393.26.5.1160 11009250

[B17] Hu X. Batchelder W. H. (1994). The statistical analysis of general processing tree models with the EM algorithm. *Psychometrika* 59 21–47. 10.1007/BF02294263

[B18] Kvavilashvili L. (1998). Remembering intentions: testing a new method of investigation. *Appl. Cogn. Psychol.* 12 533–554. 10.1002/(SICI)1099-0720(1998120)12:6<533::AID-ACP538>3.0.CO;2-1

[B19] Loftus E. F. (1971). Memory for intentions: the effect of presence of a cue and interpolated activity. *Psychon. Sci.* 23 315–316. 10.3758/BF03336128

[B20] Marsh R. L. Hicks J. L. Cook G. I. Hansen J. S. Pallos A. (2003). Interference to ongoing activities covaries with the characteristics of an event-based intention. *J. Exp. Psychol. Learn. Mem. Cogn.* 29 861–870. 10.1037/0278-7393.29.5.861 14516219

[B21] Martin B. Brown N. Hicks J. (2011). Ongoing task delays affect prospective memory more powerfully than filler task delays. *Can. J. Exp. Psychol.* 65 48–56. 10.1037/a0022872 21443330

[B22] McDaniel M. A. Einstein G. O. (2000). Strategic and automatic processes in prospective memory retrieval: a multiprocess framework. *Appl. Cogn. Psychol.* 14 S127–S144. 10.1002/acp.775

[B23] Meier B. Zimmermann T. D. Perrig W. J. (2006). Retrieval experience in prospective memory: strategic monitoring and spontaneous retrieval. *Memory* 14 872–889. 10.1080/09658210600783774 16938698

[B24] Moshagen M. (2010). multiTree: a computer program for the analysis of multinomial processing tree models. *Behav. Res. Methods* 42 42–54. 10.3758/BRM.42.1.42 20160285

[B25] Park D. C. Hertzog C. Kidder D. P. Morrell R. W. Mayhorn C. B. (1997). Effect of age on event-based and time-based prospective memory. *Psychol. Aging* 12 314–327. 10.1037/0882-7974.12.2.314 9189992

[B26] Pavawalla S. Schmitter-Edgecombe M. Smith R. E. (2012). Prospective memory after moderate-to-severe traumatic brain injury: a multinomial modeling approach. *Neuropsychology* 26 91–101. 10.1037/a0025866 21988127 PMC3271186

[B27] Scullin M. K. McDaniel M. A. Einstein G. O. (2010a). Control of cost in prospective memory: evidence for spontaneous retrieval processes. *J. Exp. Psychol. Learn. Mem. Cogn.* 36 190–203. 10.1037/a0017732 20053054

[B28] Scullin M. K. McDaniel M. A. Shelton J. T. Lee J. H. (2010b). Focal/nonfocal cue effects in prospective memory: monitoring difficulty or different retrieval processes? *J. Exp. Psychol. Learn. Mem. Cogn.* 36 736–749. 10.1037/a0018971 20438269 PMC2864946

[B29] Smith R. E. (2003). The cost of remembering to remember in event-based prospective memory: investigating the capacity demands of delayed intention performance. *J. Exp. Psychol. Learn. Mem. Cogn.* 29 347–361. 10.1037/0278-7393.29.3.347 12776746

[B30] Smith R. E. (2008). “Connecting the past and the future: attention, memory, and delayed intentions,” in *Prospective Memory. Cognitive, Neuroscience, Developmental, and Applied Perspectives* eds Kliegel M. McDaniel M. A. Einstein G. O. (London: Erlbaum) 29–52.

[B31] Smith R. E. Bayen U. J. (2004). A multinomial model of event-based prospective memory. *J. Exp. Psychol. Learn. Mem. Cogn.* 30 756–777. 10.1037/0278-7393.30.4.756 15238021

[B32] Smith R. E. Bayen U. J. (2005). The effects of working memory resource availability on prospective memory: a formal modeling approach. *Exp. Psychol.* 52 243–256. 10.1027/1618-3169.52.4.243 16304724

[B33] Smith R. E. Bayen U. J. (2006). The source of adult age differences in event-based prospective memory: a multinomial modeling approach. *J. Exp. Psychol. Learn. Mem. Cogn.* 32 623–635. 10.1037/0278-7393.32.3.623 16719671

[B34] Smith R. E. Hunt R. R. McVay J. C. McConnell M. D. (2007). The cost of event-based prospective memory: Salient target events. *J. Exp. Psychol. Learn. Mem. Cogn.* 33 734–746. 10.1037/0278-7393.33.4.734 17576150

[B35] Smith R. E. Persyn D. Butler P. (2011). Prospective memory, personality, and working memory: a formal modeling approach. *Z. Psychol.* 219 108–116. 10.1027/2151-2604/a000055 21822501 PMC3148583

[B36] Stahl C. Klauer K.-C. (2007). HMMTree: a computer program for latent-class hierarchical multinomial processing tree models. *Behav. Res. Methods* 39 267–273. 10.3758/BF03193157 17695354

[B37] Wixted J. T. Ebbeson E. B. (1991). On the form of forgetting. *Psychol. Sci.* 2 409–415. 10.1111/j.1467-9280.1991.tb00175.x

